# Supplementary material for: Venomix: a simple bioinformatic pipeline for identifying and characterizing toxin gene candidates from transcriptomic data
Source: PeerJ. 2018 Jul 31;6:e5361. doi: 10.7717/peerj.5361 (PMC6074769; doi:10.7717/peerj.5361)
Supplement: Supplemental Information 3 [file peerj-06-5361-s003.gz › FinalOutput_E-6/Acetylcholinesterase_1/finaltree.pdf]

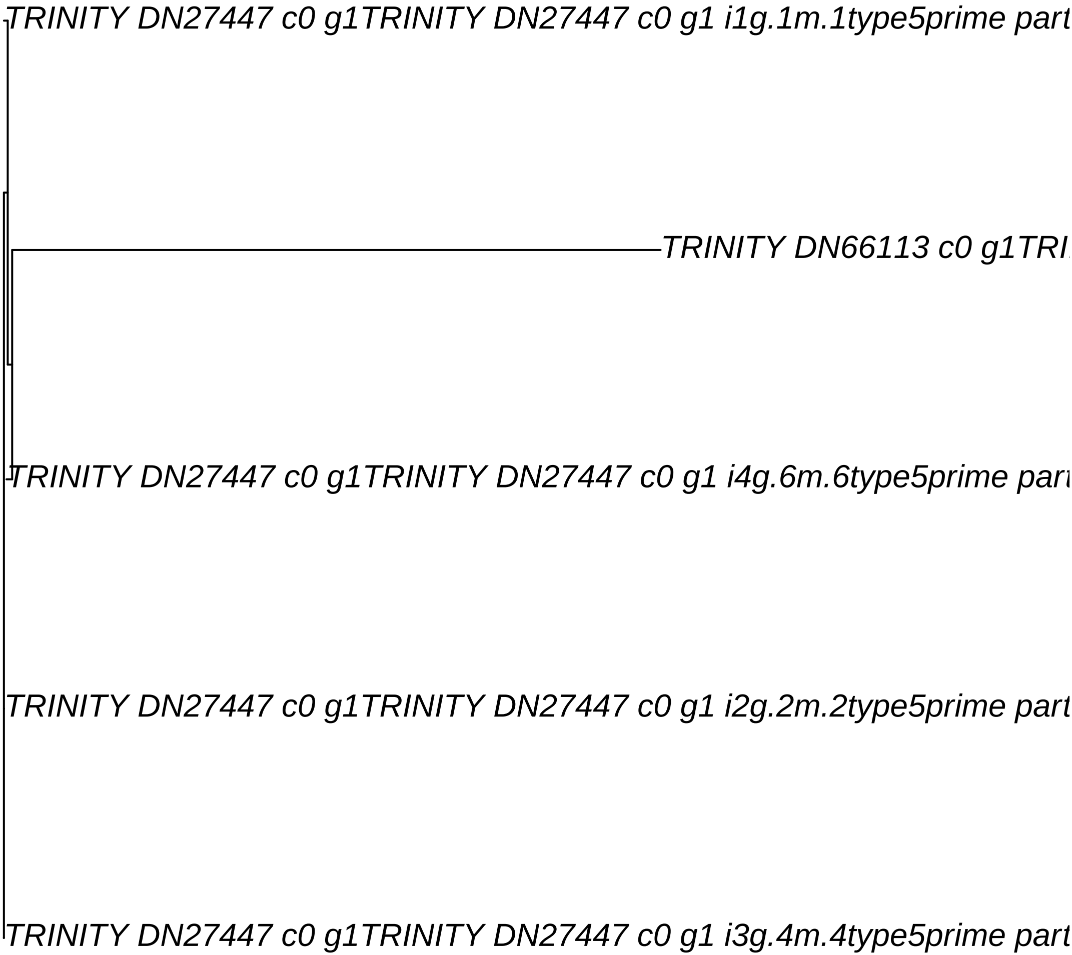
TRINITY DN27447 c0 g1TRINITY DN27447 c0 g1 i1g.1m.1type5prime part
TRINITY DN66113 c0 g1TRI
TRINITY DN27447 c0 g1TRINITY DN27447 c0 g1 i4g.6m.6type5prime part
TRINITY DN27447 c0 g1TRINITY DN27447 c0 g1 i2g.2m.2type5prime part
TRINITY DN27447 c0 g1TRINITY DN27447 c0 g1 i3g.4m.4type5prime part
